# Risk factors for coronary artery calcification in Chinese patients undergoing maintenance hemodialysis: a meta-analysis

**DOI:** 10.1007/s11255-025-04535-w

**Published:** 2025-05-02

**Authors:** Mengjiao Li, Yuxia Li, Shuting Liu, Yan Yang, Ping Jiang

**Affiliations:** 1 Shanghai University of Traditional Chinese Medicine, No. 1200 Cailun Road, Pudong New Area, Shanghai, 201203 China; 2 Chinese Medicine School of Nursing, Shanghai University of Traditional, No. 1200 Cailun Road, Pudong New Area, Shanghai, 201203 China; 3 Shanghai Pudong New District People’s Hospital, No. 490, South Chuanhuan Road, Pudong New Area, Shanghai, 200120 China

**Keywords:** Maintenance hemodialysis, Coronary artery calcification, Risk factors, Meta-analysis

## Abstract

**Background:**

Chronic kidney disease (CKD) progression to end-stage renal disease (ESRD) increases cardiovascular disease (CVD) risk, with coronary artery calcification (CAC) affecting 70% of Chinese maintenance hemodialysis (MHD) patients. Prolonged MHD treatment is linked to calcium-phosphorus imbalance and accelerated CAC progression. However, conflicting findings on CAC risk factors persist due to methodological heterogeneity in existing studies.

**Objective:**

To systematically analyze the risk factors for coronary artery calcification in patients undergoing maintenance hemodialysis in China.

**Methods:**

We conducted a computer-assisted search of ten databases, including PubMed, Web of Science, CBM, Wanfang, CNKI, and VIP, for observational studies (cohort, case–control, and cross-sectional studies) published from inception to October 21, 2024, on risk factors for coronary artery calcification (CAC) in Chinese patients with maintenance hemodialysis (MHD). The studies were independently screened by two investigators according to the PRISMA guidelines and diagnosed with coronary artery calcification through imaging techniques (CT). Odds ratios (*OR*s) and 95% confidence intervals (CIs) were used for the reported outcomes. The quality of the studies was assessed using the Newcastle–Ottawa Scale. A meta-analysis of the included data was performed using either a random effects model or a fixed effects model, we performed a meta-analysis using the Stata 17.0 software.

**Results:**

The review included 24 studies with a total sample size of 2,875 patients. A meta-analysis of these 24 studies (*n* = 2875) found that diabetes mellitus (*OR* = 2.32, 95% CI 1.37–3.27) and elevated iPTH (*OR* = 1.59, 95% CI 1.21–1.96) were the strongest predictors of an increased risk of coronary artery calcification (CAC) in Chinese maintenance hemodialysis (MHD) patients. Additionally, elevated high-sensitivity C-reactive protein (hs-CRP) levels, elevated serum calcium, advanced age, longer duration of dialysis treatment, elevated serum phosphorus, and elevated sclerostin (SOST) levels were significant predictors of an increased risk of concurrent CAC in Chinese MHD patients. However, the correlation between hypertension, serum magnesium, fibroblast growth factor-23 (FGF-23), alkaline phosphatase (ALP), and MHD with concomitant CAC was not significant, likely due to the wide variation in sample size and study type.

**Conclusion:**

Diabetes mellitus and elevated iPTH are the most significant clinical risk factors for coronary artery calcification (CAC) in Chinese maintenance hemodialysis (MHD) patients, and healthcare professionals should prioritize this population. Additionally, monitoring calcium and phosphorus metabolism, along with inflammatory markers (e.g., hs-CRP, SOST), can further reduce the risk of cardiovascular disease. Early health education and targeted, individualized treatment strategies for high-risk groups are recommended to prevent and delay the onset and progression of CAC in MHD patients.

**Supplementary Information:**

The online version contains supplementary material available at 10.1007/s11255-025-04535-w.

## Introduction

In recent years, the incidence of chronic kidney disease (CKD) has been rising, largely due to lifestyle changes, environmental factors, and inappropriate medication use. Over time, CKD inevitably progresses to end-stage renal disease (ESRD), a severe condition marked by irreversible kidney dysfunction. An estimated 132 million individuals in China have CKD, with approximately 2% progressing to ESRD annually. The five-year survival rate for ESRD patients remains critically low at 39% [1, 2]. As of December 2022, the number of individuals undergoing dialysis in China had exceeded one million. Of these patients, approximately 84.3% were receiving hemodialysis as their primary treatment modality [3]. Maintenance hemodialysis (MHD) remains a cornerstone of clinical management for patients with ESRD, serving as an effective method for toxin removal and homeostatic regulation [4]. However, prolonged hemodialysis has been shown to contribute to several adverse complications, including calcium-phosphorus metabolism disorders, hypertension, and an increased risk of cardiovascular disease (CVD), all of which can significantly impair patients' quality of life and may ultimately be life-threatening [5]. Among these complications, CVD is a major contributor to morbidity and mortality in MHD patients [6]. Notably, coronary artery calcification (CAC) is the most prevalent cardiovascular complication among Chinese hemodialysis patients, affecting approximately 70% of this population [7]. This high prevalence may be attributed to factors such as distinctive dietary patterns, the structure of the healthcare delivery system, and genetic predispositions within the Chinese population, which contribute to a risk profile for CAC that may differ from that observed in other countries. Furthermore, dialysis patients with concomitant CAC had a 1.31-fold higher risk of cardiovascular mortality compared to those without CAC [8]. Although previous studies have explored the relationship between coronary artery calcification (CAC) and dialysis patients, their findings remain highly inconsistent due to regional variations, differing study durations, and methodological heterogeneity. This meta-analysis aims to identify high-risk patients across different populations by synthesizing existing evidence—particularly through subgroup analyses—and to provide a robust evidence base for the development of targeted intervention strategies.

### Registration

This meta-analysis is registered with PROSPERO under the registration number CRD42024606240.

## Research design and methodology

### Literature search strategy

Ten electronic databases were queried: PubMed, Web of Science, CBM, EBSCO, Cochrane Library, Embase, Ovid, Wanfang, CNKI, and VIP, utilizing a computerized search system. Google Scholar served as the source of gray literature in this study. To enhance the comprehensiveness of the search, the references of the selected articles were manually reviewed to identify additional relevant literature. The search timeframe encompassed studies published from the inception of the databases until October 21, 2024, employing a combination of subject and free-text terms to maximize both sensitivity and specificity. The subject terms included “renal dialysis,” “dialysis,” “hemodialysis,” “maintenance hemodialysis (MHD),” “vascular dialysis,” “vascular calcification,” “coronary calcification,” “coronary artery calcification,” “risk factors,” “influencing factors,” and “determinants.” (Table in S1 Appendix-S1).

### Study selection criteria

The inclusion criteria for the study were as follows: (1) Study population: Chinese adults (aged ≥ 18 years) receiving maintenance hemodialysis for ≥ 3 consecutive months; (2) Study design: Cross-sectional studies, case–control studies, or prospective/retrospective cohort studies reporting multivariable-adjusted logistic regression outcomes; (3) Diagnostic criteria: Coronary artery calcification (CAC) defined as an Agatston score ≥ 100 via electrocardiogram-gated cardiac CT or a calcification volume ≥ 1 cm^3^ by quantitative computed tomography (CT); (4) Statistical analysis: Multivariable-adjusted logistic regression analyses presenting odds ratios (ORs) with corresponding 95% confidence intervals (CIs). The exclusion criteria were as follows: (1) Studies with incomplete statistical data or unavailable full-text publications; (2) Duplicate publications; (3) Reviews, conference abstracts, animal studies, and case reports; (4) Non-Chinese or non-English language literature; (5) Studies rated as having low methodological quality, defined as a score ≤ 3 on the Newcastle–Ottawa Scale (NOS) for observational studies or ≤ 4 on the Agency for Healthcare Research and Quality (AHRQ) tool for cross-sectional studies.

### Literature screening and data extraction

All identified articles were imported into EndNote for reference management. Two researchers independently screened and extracted data from the included studies through a sequential process involving title, abstract, and full-text review. The researchers then cross-verified the extracted results, resolving any discrepancies through discussion or by consulting a third investigator. Extracted data included the following variables: first author, publication year, study location, mean age of participants, duration of dialysis, diagnostic criteria, sample size, number of patients with coronary artery calcification (CAC), influencing factors, and quality assessment. The data were organized and analyzed using Microsoft Excel.

### Evaluation of literature quality

The quality of the included studies was assessed using two standardized evaluation tools. For cross-sectional studies, the quality was evaluated using the cross-sectional study quality assessment criteria developed by the Agency for Healthcare Research and Quality (AHRQ), based on the Newcastle–Ottawa Scale (NOS) [9]. This tool includes 11 items, with a total score ranging from 0 to 11: scores of 0–3 indicate low quality, 4–7 indicate moderate quality, and 8–11 indicate high quality [10]. For cohort and case–control studies, the Newcastle–Ottawa Quality Assessment Scale (NOS), designed by the Agency for Healthcare Research and Quality, was employed [11]. This tool comprises 9 items with a total score of 0–9, where scores of 1–3 represent low quality, 4–6 moderate quality, and 7–9 high quality. Studies classified as low quality were excluded from this meta-analysis.

### Statistical analysis

A meta-analysis of the extracted data was conducted using Stata 17.0 software. OR and their corresponding 95% CI, derived from multivariable logistic regression analyses, were used as effect estimates. Heterogeneity was assessed using the *I*^2^ statistic and the Q-test. Low heterogeneity was defined as *P* ≥ 0.1 and *I*^2^ < 50%, in which case a fixed-effects model was applied. Conversely, for *P* < 0.1 or *I*^2^ ≥ 50%, indicating substantial heterogeneity, a random-effects model was employed. Sensitivity analyses, subgroup analyses, and meta-regressions were subsequently performed on factors exhibiting high heterogeneity to identify potential sources of heterogeneity. Sensitivity analyses were performed by comparing the results of the fixed-effects model with those of the random-effects model. In addition, a leave-one-out analysis was conducted, in which each study was excluded in turn to assess its influence on the overall pooled estimates. This approach was used to evaluate the robustness and consistency of the results. Subgroup analyses were conducted to explore sources of heterogeneity according to potential effect modifiers, including geographic region, publication year, and study design. Meta-regression analyses were subsequently conducted for covariates represented in ≥ 10 studies. These analyses aimed to statistically evaluate the influence of study-level characteristics on the observed effect sizes. Finally, funnel plots, Begg’s test, and Egger’s test were applied to evaluate publication bias among studies exhibiting high heterogeneity. In this study, a *P* value > 0.05 indicated relatively stable results and a low risk of publication bias, whereas a *P* value < 0.05 suggested significant publication bias.

## Results

### Literature search and screening

**Fig. 1:**
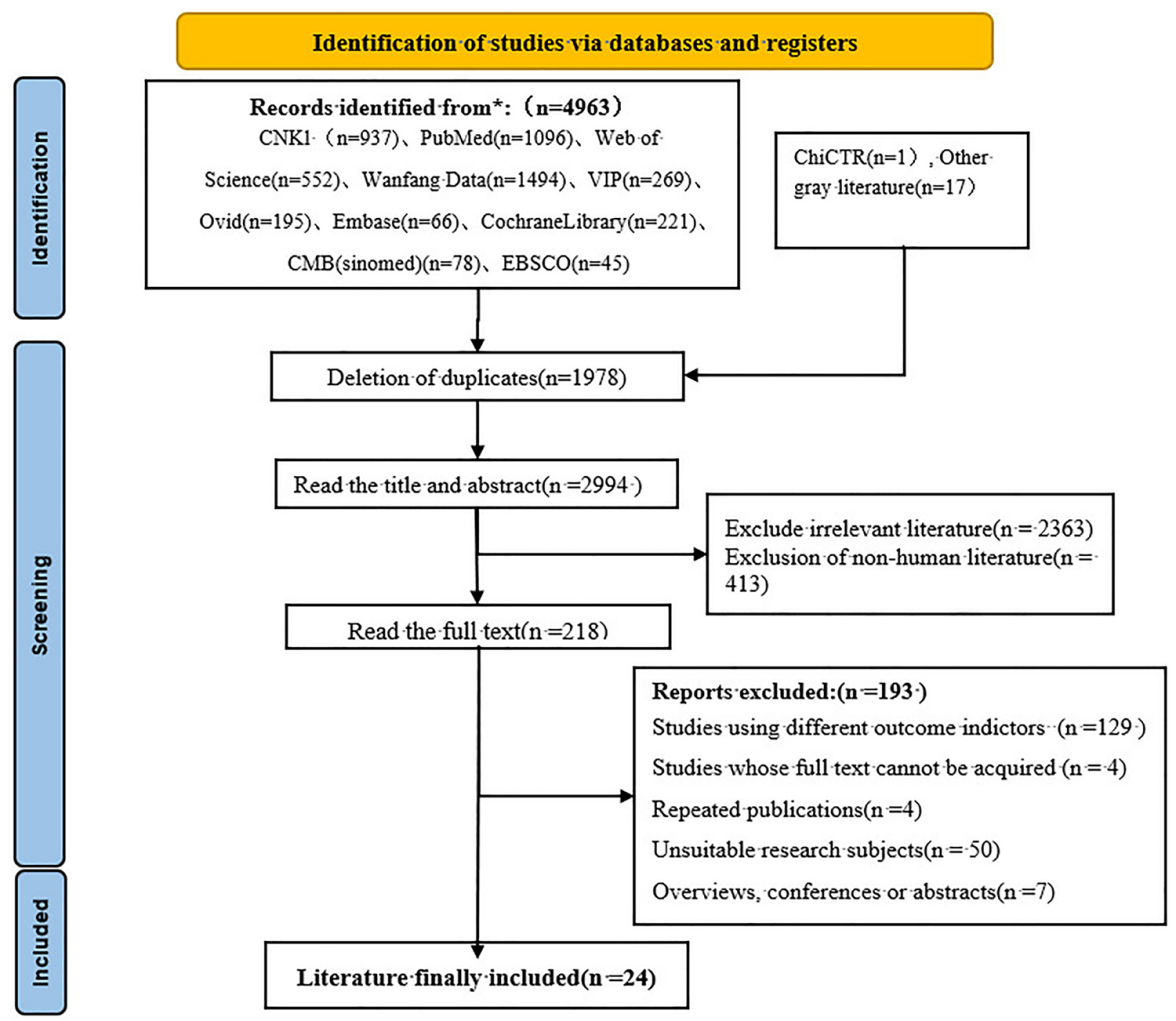
Flow chart depicting the literature

A preliminary database search identified 4,981 relevant publications. After a stepwise screening process, 24 [5, 12, 13] publications were ultimately included in the analysis. These studies included a total sample of 2875 patients, among whom 1984 cases of CAC were identified in patients undergoing hemodialysis (Fig. 1).

### Characteristics and quality assessment of the included studies

A total of 13 cross-sectional studies [13–22], 4 case–control studies [23–25], and 7 cohort studies [12, 26–30] were selected for this study. Quality assessment showed that the scores of the cohort and case–control studies were 7–9 on the Document Quality Assessment (DQA) scale and 7–10 on the DQA scale for the cross-sectional studies. A total of 36 potential risk factors were identified in these studies (Table 1).

**Table 1:** Basic characteristics of literature and quality assessment

Included literature	Year	Type of study	Duration of dialysis	Diagnostic criteria	Sample size	Influencing factors	NOS/AHRQ
Lei et al. [12]	2023	II	≥ 6	Agatston	110	13, 16, 17	8
Cheng et al. [14]	2019	I	> 3	Agatston	138	12	7
Kang et al. [23]	2023	II	> 12	Agatston	91	1, 5, 6, 9	7
Ran et al. [31]	2016	I	≥ 6	Agatston	43	6, 13	7
Zhou et al. [32]	2020	II	≥ 3	Agatston	198	3, 5, 10, 18, 19	8
He et al. [33]	2021	III	≥ 3	Agatston	98	6, 10, 12, 20	8
Cui et al. [15]	2015	I	≥ 3	Agatston	53	1, 3, 6, 21	8
Jiang et al. [26]	2020	III	≥ 3	Agatston	98	2, 6, 14, 15, 9	8
Dong et al. [16]	2024	I	≥ 3	Agatston	117	6	8
Xu et al. [27]	2021	III	≥ 3	Agatston	111	5, 22	8
Jia et al. [28]	2015	III	≥ 3	Agatston	95	1, 4, 7, 10, 13,	
Lai et al. [24]	2022	II	≥ 3	Agatston	100	1, 3, 5, 8	7
Chen et al. [5]	2020	II	≥ 3	Agatston	152	1, 4, 10, 23, 24	8
Yang et al. [17]	2017	I	≥ 3	Agatston	201	1, 2, 3, 4, 6, 25, 26	7
Cui et al. [18]	2024	II	> 6	Agatston	110	1, 5, 12, 15, 27	8
Xie et al. [29]	2022	I	≥ 6	Agatston	104	1, 9	8
Chen et al. [19]	2017	I	> 12	Agatston	131	1, 8, 13	8
Zhou et al. [20]	2024	II	≥ 3	Agatston	123	1, 3, 5, 7	7
Shao et al. [34]	2021	II	≥ 6	Agatston	91	1, 9, 28, 29	7
Fan et al. [25]	2023	II	≥ 3	Agatston	138	1, 3, 7, 10	8
Xiong et al. [21]	2024	II	≥ 3	Agatston	123	1, 5, 8, 11, 30, 31, 32, 33, 34	8
Hu et al. [22]	2022	II	> 3	Agatston	148	6, 12, 13, 35	9
Jiang et al. [30]	2021	I	≥ 3	Agatston	154	8, 1, 3, 15, 36	7
Guo et al. [13]	2023	I	≥ 6	Agatston	148	12, 1, 27, 37	9

Research type: I Cross-sectional study; II case–control study, III Cohort Study.Influencing factors include:1. Age, 2. Gender, 3. Dialysis duration, 4. Hypertension, 5. Diabetes mellitus, 6. Serum phosphorus, 7. Serum calcium, 8. Serum magnesium, 9. Parathyroid hormone (PTH), 10. High-sensitivity C-reactive protein (hs-CRP), 11. Total cholesterol, 12. sclerostin(SOST), 13. Fibroblast growth factor-23 (FGF-23), 14. Low-density lipoprotein cholesterol (LDL-C), 15. Alkaline phosphatase (ALP), 16. Fibroblast growth factor-21 (FGF-21), 17. Insulin-like growth factor (IGF), 18. Galectin-3, 19. Matrix metalloproteinase-9 (MMP-9);20. Sclerostin miRNA-29, 21. Blood pH, 22. Serum sorting protein, 23. Serum creatinine, 24. Serum zinc-α2-glycoprotein (ZAG), 25. Serum potassium, 26. Urea clearance index (Kt/V), 27. Triglycerides, 28. Smoking history, 29. Pruritus score (VAS), 30. Body mass index (BMI), 31. Calcium-phosphorus product, 32. N-MID OC, 33. β-type I collagen cross-linked C-terminal peptide (β-CrossLaps), 34. Cholesterol(TC), 35. sTWEAK, 36. Coronary heart disease, 37. Irisin

### Meta-analysis of factors influencing concurrent CAC in patients with MHD

This meta-analysis performed a comprehensive evaluation of 13 determinants, which were categorized into three domains: sociodemographic characteristics, comorbid conditions, and biochemical parameters. Socio-demographic variables comprised age [13, 21, 23, 30] (analyzed as dichotomous variable with cutoff at ≥ 60 years [15, 25, 34]) and length of dialysis treatment [32]; comorbid conditions were defined as pre-existing diabetes mellitus (DM) [23] and essential hypertension (HTN) [5] documented in medical records. Biochemical parameters quantified through standardized assays included: phosphorus (P) [23], calcium (Ca^2+^) [28], magnesium (Mg^2+^) [24], alkaline phosphatase (ALP) [26], intact parathyroid hormone (iPTH) [23], high-sensitivity C-reactive protein (hs-CRP) [20], sclerostin (SOST) [14], and fibroblast growth factor-23 (FGF-23) [12].

#### Meta-analysis results

Firstly, this meta-analysis synthesized data from multiple studies investigating various influential factors. The results revealed that sociodemographic factors, including continuous age [13, 21, 23, 30] increase (OR = 1.08, 95% CI 1.05–1.11), advanced age ≥ 60 years [15, 25, 34] (OR = 1.09, 95% CI 1.01–1.18), and length of dialysis treatment [32] (OR = 1.02, 95% CI 1.01–1.04). These parameters emerged as significant predictors of CAC progression in MHD populations. Notably, within comorbidity profiles, diabetes mellitus demonstrated the strongest association [23] (OR = 2.32, 95% CI 1.37–3.27) with coronary artery calcification progression in maintenance hemodialysis recipients. The biochemical analysis identified six significant mediators:· Serum phosphorus [23](OR = 1.18, 95% CI 1.05–1.30),calcium [28](OR = 1.36, 95% CI 1.17–1.56). iPTH [23](OR = 1.59, 95% CI 1.21–1.96),hs-CRP [20] (OR = 1.48, 95% CI 1.34–1.72),SOST [14] (OR = 1.20, 95% CI 1.01–1.38). These biomarkers showed significant dose–response relationships with vascular calcification severity in the hemodialysis cohort. Serum magnesium levels [24]demonstrated no significant association with coronary artery calcification progression in the maintenance hemodialysis cohort within the detection limits of this analysis. The 95% confidence intervals for hypertension [5], ALP [26], and FGF-23 [12]included the null value (with lower limits < 1 and upper limits > 1), therefore the null hypothesis could not be rejected (*P* > 0.05). This statistical evidence indicates these exposure variables showed no statistically significant association with the clinical outcome (Table 2, Figs in S1 Appendix S5 to S7).

**Table 2:** Heterogeneity test and meta-analysis of factors influencing concurrent CAC in MHD patients

Factor	No. of studies	Sample size	*I*^*2*^	Model used	Effect size		
					OR	95% CI	*P* value
Age	15	1814	75.8	Random-effects	1.08	(1.05,1.11)	< 0.001
Age ≥ 60 years	3	329	13.4	Fixed-effects	1.09	(1.01,1.18)	< 0.001
Duration of dialysis treatment	7	967	52.9	Random-effects	1.02	(1.01,1.04)	< 0.001
Diabetes mellitus	7	856	85.9	Random-effects	2.32	(1.37,3.27)	< 0.001
Hypertension	3	448	63	Random-effects	1.65	(0.28,3.02)	0.019
Serum phosphorus	8	831	14.8	Fixed-effects	1.18	(1.05,1.30)	< 0.001
Calcium	3	356	0	Fixed-effects	1.36	(1.17,1.56)	< 0.001
Magnesium	4	354	0	Fixed-effects	0.001	(-0.001,0.003)	0.324
iPTH	4	384	0	Fixed-effects	1.59	(1.21,1.96)	< 0.001
Hs*-*CRP	5	688	31.2	Fixed-effects	1.48	(1.34,1.72)	< 0.001
SOST	5	649	84.4	Random-effects	1.2	(1.01,1.38)	< 0.001
FGF*-*23	4	379	88.1	Random-effects	1.03	(1.00,1.06)	< 0.001
ALP	3	362	82.6	Random-effects	1.03	(1.00,1.07)	< 0.001

*iPTH* parathyroid hormone, *hs-CRP* hypersensitive C-reactive protein, *SOST* Sosteosclerotic protein, *FGF-23* fibroblast growth factor-23, *ALP* alkaline phosphatase

#### Subgroup analyses and meta-regression

In this study, subgroup analyses were conducted for factors exhibiting high heterogeneity, and meta-regression analyses were performed for factors with ≥ 10 publications. The results of subgroup analyses showed higher heterogeneity in longitudinal studies regarding age, greater heterogeneity in cross-sectional and longitudinal studies for dialysis treatment duration and FGF-23, and greater heterogeneity in cross-sectional and case–control studies for diabetes when analyzed from the perspective of study type; however, subgroup analyses of case–control studies regarding dialysis treatment duration did not yield a statistically significant effect. Analyzed from the perspective of the study region, heterogeneity was greater in eastern China for age, SOST, and FGF-23, and in Western China for dialysis treatment duration and diabetes. Analyzed from the perspective of the year of publication, heterogeneity was higher for age, dialysis treatment duration, diabetes, FGF-23, and hypertension between 2015 and 2020. Between 2021 and 2024, heterogeneity was higher for dialysis treatment duration, SOST, and ALP. Meta-regression analysis of the factor 'age' in studies with ≥ 10 publications showed [OR = 0.13, 95% CI (− 0.05, 0.32), SE = 0.09, *t* = 1.56, *P* = 0.146]. However, this analysis did not identify any source of heterogeneity related to age (Fig in S1 Appendix-S8).

#### Results of descriptive analysis

In addition to the aforementioned factors, several other variables were identified that did not exhibit a significant correlation with concomitant CAC in MHD patients. These factors include blood creatinine, ZAG [5], sclerostin miRNA-29b [33], FGF-21, IGF-1 [12], Gal-3, MMP-9 [32], LDL [26], pH [15], serum sorting protein [27], Kt/V, serum potassium(K) [17], TyG [18], smoking, pruritus score [34], BMI, calcium-phosphorus product, N-MID OC, β type I collagen cross-linked carboxy-terminal peptide (beta-CTX), cholesterol(TC) [21], sTWEAK [22], coronary heart disease [30], and irisin [13].

#### Sensitivity analysis and publication bias

##### Sensitivity analysis

In this study, sensitivity analyses were conducted for factors exhibiting high heterogeneity. Notably, combined diabetes mellitus and hypertension demonstrated substantial differences in results when changing the effect model, while age, hypertension, SOST, and FGF-23 showed consistent results across both models. This suggests that the results for age, dialysis duration, SOST, ALP, and FGF-23 were more stable (Figs in S1 Appendix S9). Additionally, sensitivity analysis was performed using a one-by-one exclusion method for factors with high heterogeneity. For example, excluding the study by Jia et al. [28] on age as a comorbid factor, statistical heterogeneity among the remaining 12 studies was eliminated (*I*^2^ = 0%, *P* < 0.001). Using a fixed-effects model, age was confirmed as a risk factor for CAC in Chinese patients on MHD [OR = 1.07, 95% CI (1.06,1.08), *Z* = 158.17, *P* = 0.506]. Similarly, after excluding the study by Lai et al. [24] on diabetes, the remaining six studies showed no statistical heterogeneity (*I*^2^ = 48.4%, *P* < 0.001). The fixed-effects model indicated that diabetes was a risk factor for CAC in Chinese MHD patients [OR = 1.3, 95% CI (1.10–1.49), *Z* = 12.98, *P* = 0.085]. Excluding He et al. [33] for SOST, no heterogeneity was observed in the remaining studies (*I*^2^ = 0%, *P* < 0.001), and the fixed-effects model showed that SOST was a risk factor for CAC in Chinese MHD patients [OR = 1.25, 95% CI (1.15,1.35), *Z* = 24.51, *P* = 0.415]. Regarding hypertension, excluding the study by Yang et al. [17] significantly reduced heterogeneity (*I*^2^ = 0%, *P* < 0.001). The fixed-effects model demonstrated that hypertension was a risk factor for CAC in Chinese MHD patients [OR = 2.56, 95% CI (1.21–3.91), *Z* = 3.73, *P* = 0.545]. Finally, excluding Jiang et al. [30] for ALP resulted in no statistical heterogeneity among the remaining studies (*I*^2^ = 44.5%, *P* < 0.001), and a fixed-effects model confirmed that ALP was a risk factor for CAC in Chinese MHD patients [OR = 1.05, 95% CI (1.02,1.07), *Z* = 83.6, *P* = 0.179]. For other influencing factors, heterogeneity remained unchanged with both the one-by-one exclusion method and the effect model alteration.

##### Publication bias

In this study, publication bias was assessed using Egger's test, Begg's test, and funnel plots, based on influencing factors with literature inclusion of ≥ 2 articles. The results indicated potential publication bias for age (*P* = 0.01), dialysis duration (*P* < 0.001), blood phosphorus (*P* = 0.001), and iPTH (*P* = 0.031), as a risk of bias is considered present when the *P*-value is < 0.05. Therefore, it is suggested that publication bias may exist for these factors, while no significant publication bias was detected for the other influencing factors (Figs in S1 Appendix S10 to S11).

## Discussion

### General factors affecting complicated CAC in patients with MHD

#### Socio-demographic factors

The results of this study indicated that increasing age is a significant risk factor for the development of CAC in MHD patients. This finding aligns with the results reported by Hui et al. [35]. The increased risk associated with age may be attributed to the natural decline in body functions over time, which accelerates the degradation of vascular elasticity and slows hemodynamic velocity. These age-related changes can lead to increased intravascular calcium salt deposition, subsequently increasing the risk of endovascular and valve calcification. Long-term dialysis patients are more prone to CAC due to the accumulation of large molecular toxins and calcium and phosphorus imbalances that are not effectively eliminated by dialysis. These imbalances lead to the deposition of these substances in the vessels. Additionally, long-term dialysis causes endothelial damage and sustained systemic inflammation, both of which contribute to the progression of vascular calcification [36]. Moreover, patients are often required to take calcium supplements to maintain calcium balance during dialysis; however, excessive calcium supplementation can result in blood calcium deposition, further exacerbating the risk of CAC [37]. Considering these findings, it is essential to identify and screen elderly male patients with long dialysis durations for relevant risk factors as early as possible. Timely intervention should be considered to manage these risk factors and reduce the risk of CAC progression.

#### Disease-related factors

The findings of this study suggest that patients with comorbid diabetes are at an increased risk of developing CAC. This study[38, 39] demonstrated that the incidence of concomitant CAC in MHD patients with diabetic nephropathy was twice that of MHD patients with nondiabetic nephropathy, and their survival rate was significantly lower. Chronic hyperglycemia can induce vascular endothelial damage, reduce vascular elasticity, and promote calcium-phosphorus deposition in the bloodstream, ultimately leading to increased vascular calcification. Moreover, diabetes itself contributes to vascular calcification through multiple pathways [40]. For instance, hyperglycemia promotes an increase in osteoblasts and ALP. In vitro, vascular smooth muscle cells (VSMCs) exposed to a hyperglycemic environment express elevated levels of osteoclast transcription factors, which subsequently enhance the Wnt signaling pathway [41, 42], leading to increased osteoblast activity [43]. Furthermore, calcium-phosphorus and hydroxyapatite crystal deposition in the vascular endothelium further exacerbates arterial calcification [44]. These findings underscore the complex and multifactorial role of diabetes mellitus in vascular calcification and CAC development in MHD patients. Moreover, prolonged hyperglycemia accelerates these pathological processes, necessitating stringent blood glucose management in diabetic MHD patients to prevent or mitigate CAC progression. The association between hypertension and concomitant CAC in MHD patients is not statistically significant. However, hypertension induces activation of the vascular wall renin-angiotensin system (RAS) through mechanical stress, facilitating calcium-phosphorus deposition. This process leads to VSMC damage and dysfunction, ultimately exacerbating CAC development [45]. However, the high prevalence of vascular sclerosis and blood pressure fluctuations in long-term dialysis patients may confound the linear relationship between blood pressure and CAC. Moreover, increased arterial stiffness due to vascular calcification may paradoxically distort blood pressure measurements, thereby weakening the observed correlation [46].

### Physiologic indicators affecting complicated CAC in patients with MHD

The results of this study demonstrate that elevated serum phosphorus and calcium levels, increased secretion of iPTH, elevated hs-CRP, and upregulated SOST are key contributors to the heightened risk of concomitant CAC in MHD patients. Hyperphosphatemia contributes to CAC development through multiple mechanisms: Elevated serum phosphorus directly stimulates parathyroid hormone (PTH) secretion, promotes osteoblast differentiation, and facilitates pathological calcium-phosphorus deposition in the vascular wall [47]. Excess phosphorus induces the transdifferentiation of VSMCs into osteoblast-like cells, exacerbates local inflammatory responses, and accelerates calcified plaque formation via activation of the NF-κB pathway [48] Additionally, excess phosphorus dysregulates FGF-23 levels, further contributing to vascular endothelial dysfunction. Clinicians should closely monitor dynamic changes in serum phosphorus levels, enhance comprehensive management, adhere to the "3D principle" [49], and implement personalized dialysis strategies for MHD patients. Long-term dialysis has been shown to promote calcium ion deposition in the bloodstream, leading to calcium salt accumulation in the vascular wall, which directly damages endothelial cells and induces vascular medial calcification [36]. This process has been shown to stimulate secondary hyperparathyroidism (SHPT), resulting in increased calcium release from bones into the bloodstream. Consequently, this exacerbates hypercalcemia and further accelerates vascular calcification via inflammatory pathways. Furthermore, calcium supplementation is often necessary to maintain calcium homeostasis during dialysis. However, excessive calcium supplementation may contribute to pathological calcium deposition in the vasculature [37]. The present study demonstrates that excessive synthesis and secretion of iPTH significantly increase the risk of concomitant CAC in patients with MHD. Observational studies indicate that MHD patients with iPTH levels exceeding 300 pg/mL exhibit an approximately 2.5-fold increase in CAC prevalence compared to those with iPTH levels in the 150–300 pg/mL range [50]. Additionally, studies have reported a 35% increase in Agatston scores for every 100 pg/mL elevation in iPTH levels [51]. Excessive iPTH synthesis and secretion disrupt bone and calcium-phosphorus metabolism, promotes pathological calcium-phosphorus deposition in dialysis patients, and stimulate VSMC differentiation and transdifferentiation [52]. Additionally, iPTH can directly induce the expression of chondrogenic markers in vascular endothelial cells, promoting their differentiation into chondrocyte-like cells, ultimately contributing to vascular calcification [53]. According to the KDIGO guidelines, therapeutic strategies for managing elevated iPTH levels include restricting calcium and phosphorus intake through the use of phosphate binders, calcimimetics, and active vitamin D or its analogs [54]. However, clinical studies indicate that only 45% of dialysis centers conduct monthly iPTH testing, which falls significantly short of the KDIGO-recommended quarterly assessments [55]. This discrepancy partly stems from inadequate patient education on phosphorus-restricted diets and the underuse of phosphate binders. Therefore, regular training for clinical caregivers on calcium and phosphorus metabolism management is essential, with a focus on individualized dosing of active vitamin D and calcimimetics. The use of mobile applications to support personalized dietary plans (e.g., low-phosphorus meal planning) and medication adherence reminders is also recommended to improve patient compliance. Elevated hs-CRP levels have been shown to promote the release of interleukin-6 (IL-6) and endothelin [50]. IL-6 binds to its receptor, leading to the activation of phosphorylated signal transducer and activator of transcription 3 (p-STAT3). This subsequently upregulates the expression of runt-related transcription factor 2 (RUNX2), alkaline phosphatase, and osteoprotegerin (OPN), thereby promoting the transdifferentiation and calcification of VSMCs [56]. Secreted SOST regulates bone metabolism and contributes to CAC through the Wnt/β-catenin signaling pathway [57].

### Factors not related to complicated CAC in patients with MHD

The findings of this study indicated that serum magnesium was not associated with CAC in MHD patients, nor were hypertension, FGF-23 growth, and ALP significantly linked to CAC. However, previous studies have indicated that the prevalence of CAC is 1.4 times higher in MHD patients with comorbid hypertension [58]. This phenomenon can be attributed to the fact that hypertension can induce activation of the renin-angiotensin system (RAS) in the vascular wall through mechanical forces. This, in turn, promotes calcium and phosphorus deposition in the blood, leading to vascular endothelial cell damage and dysfunction, thereby exacerbating CAC [45]. In the context of long-term dialysis patients, the vascular wall becomes stiffer and less elastic due to aberrations in calcium and phosphorus metabolism. These patients also suffer from hypertension or hypotension triggered by water and sodium retention and the removal of excess fluid during dialysis, as well as uremic toxin accumulation and the presence of a microinflammatory state. These factors damage the vascular endothelium and promote calcification and atherosclerosis [59]. Not only can all of these factors potentially confound the linear relationship between blood pressure (BP) and CAC, but arterial stiffness resulting from vascular calcification can also introduce measurement errors in BP, thereby significantly reducing the correlation in the results. Low serum magnesium is an independent risk factor for mortality in MHD patients with comorbid cardiovascular disease [60, 61]. Additionally, blood magnesium competes with calcium ions in hydroxyapatite, reducing crystal stability and increasing solubility, thereby inhibiting calcium salt deposition in the vasculature. Magnesium slow the process of vascular calcification by inhibiting hydroxyapatite crystal formation, blocking the conversion of VSMCs to osteoblast-like cells, and downregulating pro-calcitonin [62]. Additionally, magnesium inhibits the osteogenic transformation of VSMCs by suppressing the Wnt/β-catenin pathway, leading to the downregulation of Run-related transcription factor 2 (Runx2) and osteoprotegerin (OPN) expression [63]. Furthermore, several studies have shown that dialysis patients with serum magnesium levels < 1.15 mmol/L have a significantly increased risk of coronary artery calcification (CAC) and are negatively associated with all-cause mortality [64, 65]. Furthermore, FGF-23 [66] and ALP [67] have been shown to directly elevate the risk of concomitant CAC in MHD patients.FGF-23 can promote the transdifferentiation of VSMCs into osteoblast-like cells via direct activation of the Wnt/β-catenin signaling pathway, leading to the formation of calcified nodules that adhere to the vessel wall and further exacerbate vascular calcification [68]. Elevated ALP facilitates vascular calcification by hydrolyzing pyrophosphate into inorganic phosphate, thereby reducing pyrophosphate levels and promoting mineral deposition [69, 70]. Therefore, in MHD patients with hypomagnesemia, dynamic monitoring of serum magnesium levels should be performed in conjunction with urinary magnesium excretion to accurately assess magnesium status, particularly in patients with diabetes or those receiving diuretics. However, careful interpretation of these measurements is required. Continuous non-invasive arterial pressure (CNAP) monitoring can be utilized to evaluate vascular calcification burden in hypertensive patients during clinical management. This approach aids in optimizing antihypertensive drug selection, with a preference for agents that confer additional vasoprotective effects. Additionally, FGF23 has a molecular weight of approximately 32 kDa. High-flux hemodialysis (HF-HD) is widely utilized in clinical practice for dialysis treatment, facilitating the removal of various uremic toxins and reducing their accumulation in the body.

### Limitations

The present study encountered heterogeneity in the types of literature included. Initially, the limited number of studies on hypertension, serum magnesium, FGF-23, and ALP in the included literature may be attributed to language barriers, leading to an inadequate sample size. Consequently, these factors did not show significant associations with concomitant CAC in MHD patients, nor did they align with the findings of other studies. Secondly, the sample sizes of the included studies varied considerably for key factors, including hypertension, serum magnesium levels, and FGF-23, leading to 95% CI that included the value 1. This, in turn, introduced uncertainty in the results. Finally, the presence of multiple confounding factors and publication bias, likely due to substantial discrepancies in sample sizes and study heterogeneity, further complicates the result interpretation. Based on these findings, future meta-analyses should incorporate a larger number of high-quality, original studies with extensive sample sizes and diverse populations. Furthermore, given the potential role of hypertension, serum magnesium, FGF-23, and ALP in increasing disease risk, future research should prioritize these factors in subsequent analyses to further validate and substantiate existing findings.

## Summary

In summary, this study concludes that advanced age, prolonged dialysis duration, concomitant diabetes, hypertension, hyperparathyroidism, and elevated levels of PTH and hs-CRP are significant risk factors for CAC in patients with ESRD. The meta-analysis results highlight advanced age, prolonged dialysis duration, concomitant diabetes, hypertension, hyperparathyroidism, and elevated levels of CRP, hs-CRP, and SOST as key risk factors for CAC in ESRD patients. Notably, diabetes and elevated PTH levels are particularly significant risk factors for CAC progression. Therefore, Clinical strategies should focus on intensive diabetes management (target HbA1c < 7.0%), hypertension control (< 130/80 mmHg) using renin-angiotensin inhibitors and fluid management to prevent endothelial dysfunction. Hyperparathyroidism should be managed by PTH levels (150–300 pg/mL) with calcimimetics and active vitamin D analogs. Inflammation can be mitigated by monitoring hs-CRP levels and considering anti-inflammatory treatments such as statins and IL-6 inhibitors. Multidisciplinary care should involve nephrologists, endocrinologists, and dietitians in developing personalized calcium-phosphorus management strategies, including phosphate binders and dietary restrictions, along with regular CAC screening using CACS.

## Supplementary Information

Below is the link to the electronic supplementary material.

Supplementary file1 (DOCX 48 KB)

Supplementary file2 (DOCX 34 KB)

Supplementary file3 (DOCX 671 KB)

## Data Availability

The datasets used and/or analyzed in this study are available upon request from the corresponding author. All data generated or analyzed during this study are included in this published article and its supplementary information files

## References

[1] Bikbov B, Purcell CA, Levey AS et al, on behalf of the GBD Chronic Kidney Disease Collaboration (2020) Global, regional, and national incidence, prevalence, and years lived with disability for chronic kidney disease from 1990 to 2017: a systematic analysis for the Global Burden of Disease Study 2017. Lancet 395:709–733. 10.1016/S0140-6736(20)30134-9 10.1016/S0140-6736(20)30045-3 PMC7049905 32061315

[2] Yang C, Zhang L, Zhao MH (2023) Interpretation of the guidelines for early evaluation and management of chronic kidney disease in China. Chin J Pract Internal Med 43(10):839–841. 10.19538/j.nk2023100109

[3] Chinese Medical Doctor Association Nephrology Branch 2023 Annual Academic Conference. Chinese Medical Doctor Association Nephrology Branch, Qingdao (2023). 10.3969/j.issn.1006-298X.2023.01.001

[4] Liyanage T, Ninomiya T, Jha V et al (2015) Worldwide access to treatment for end-stage kidney disease: a systematic review. Lancet 385(9981):197–207. 10.1016/S0140-6736(14)61601-9 10.1016/S0140-6736(14)61601-9 25777665

[5] Chen Z, Gao LP, Su H (2020) The diagnostic value of serum zinc-α2-glycoprotein in coronary artery calcification in patients undergoing maintenance hemodialysis. J Cardiovasc Pulm Dis 39(10):5. 10.3969/j.issn.1007-5062.2020.10.011

[6] Kraus MA, Kalra PA, Hunter J et al (2012) The prevalence of vascular calcification in patients with end-stage renal disease on hemodialysis: a cross-sectional observational study. Ther Adv Chronic Dis. 10.1177/204062231557865 10.1177/2040622315578654 PMC4416967 25984289

[7] Liu ZH, Yu XQ, Yang JW et al (2018) Prevalence and risk factors for vascular calcification in Chinese patients receiving dialysis: baseline results from a prospective cohort study. Curr Med Res Opin. 10.1080/03007995.2018.1467886 29672176 10.1080/03007995.2018.1467886

[8] Zhang J, Pang Q, Wang S et al (2023) Associated factors ofcardiac valve calcification and its prognostic effects amongpatients with chronic kidney disease:a systematic reviewand meta-analysis. Front Cardiovasc Med 10:1120634. 10.3389/fcvm.2023.1120634 37180797 10.3389/fcvm.2023.1120634 PMC10169583

[9] Zeng XT, Liu H, Chen X et al (2012) Meta-analysis series IV: quality assessment tools for observational studies. Chin J Evid Based Cardiol 4(4):3. 10.3969/j.1674-4055.2012.04.004

[10] Prins J, Blanker MH, Bohnen AM et al (2003) Prevalence of erectile dysfunction: a systematic review of population-based studies. Int J Impot Res 14(6):422–432. 10.1038/sj.ijir.3900905 10.1038/sj.ijir.3900905 12494273

[11] Stang A (2010) Critical evaluation of the Newcastle-Ottawa scale for the assessment of the quality of nonrandomized studies in meta-analyses. Eur J Epidemiol 25(9):603–605. 10.1007/s10654-010-9491-z 20652370 10.1007/s10654-010-9491-z

[12] Lei AL, Liu WY, Zhao JT et al (2023) Correlation between serum fibroblast growth factors 23, 21, and insulin-like growth factor 1 with coronary artery calcification and cardiovascular events in patients undergoing maintenance hemodialysis. Chin J Mod Med 25(12):44–48. 10.3969/j.issn.1672-9463.2023.012.009

[13] Guo W, Xiong L, Wu W et al (2023) Screening and correlation analysis of serum markers related to coronary artery calcification in maintenance hemodialysis patients. Chin J Arterioscler 31(4):336–342. 10.20039/j.cnki.1007-3949.2023.04.008

[14] Cheng J, Wang W, Liu GX (2019) Correlation study of serum osteocalcin with coronary artery calcification in patients undergoing maintenance hemodialysis. J Clin Nephrol 19(2):86–90. 10.3969/j.issn.1671-2390.2019.02.002

[15] Cui LW, Xu JS, Bai YL et al (2015) Factors influencing coronary artery calcification in patients with end-stage renal disease. Chin Med J 95(38):3133–3137. 10.3760/cma.j.issn.0376-2491.2015.38.015 26814105

[16] Dong LP, Liu S, Xu Q et al (2024) Correlation between peripheral blood miR-125b-5p and coronary artery calcification in patients undergoing maintenance hemodialysis. Chin J Mol Cardiol 24(1):5844–5852. 10.16563/j.cnki.1671-6272.2024.02.003

[17] Yang LL, Xing L, Cheng Y et al (2017) Risk factors for coronary artery calcification in hemodialysis patients. Int J Transpl Hemodial 15(3):13–16. 10.3760/cma.j.issn.1673-4238.2017.03.an003

[18] Cui L, Shi GC, Su C et al (2024) Predictive value of serum SOST, ALP levels, and tyg index for coronary artery calcification in patients undergoing maintenance hemodialysis. J Mol Diagn Therapy 16(2):239–242, 246. 10.3969/j.issn.1674-6929.2024.02.010

[19] Chen Y, Zheng SP, Jin LW et al (2017) Correlation analysis between magnesium metabolism disorders and coronary artery calcification in patients undergoing maintenance hemodialysis. Chin J Nephrol 33(2):106–111. 10.3760/cma.j.issn.1001-7097.2017.02.005

[20] Zhou X, Gan H (2024) Correlation between acid-base environment and coronary artery calcification in patients undergoing maintenance hemodialysis. Front Med Sci Res. 10.25236/fmsr.2024.060408

[21] Xiong L, Chen QQ, Cheng Y et al (2024) The relationship between coronary artery calcification and bone metabolic markers in patients undergoing maintenance hemodialysis. BMC Nephrol 24(1):238. 10.1186/s12882-023-03286-z 10.1186/s12882-023-03286-z PMC10428586 37582785

[22] Hu X, You X, Qu X et al (2022) Correlation between FGF-23, sclerostin, sTWEAK and coronary artery calcification in maintenance hemodialysis patients. J Mol Diagn Ther 14(5):840–843. 10.19930/j.cnki.jmdt.2022.05.020

[23] Kang X (2023) Analysis of factors affecting coronary artery calcification in elderly patients undergoing maintenance hemodialysis. Knowl Cardiovasc Dis Prev Treat 13(13):13–15. 10.3969/j.issn.1672-3015(x).2023.13.005

[24] Lai WJ, Wang SQ, Jiang YB et al (2022) Risk factors for coronary artery calcification in elderly patients undergoing maintenance hemodialysis. Western Med 34(6):882–886. 10.3969/j.issn.1672-3511.2022.06.021

[25] Fan H, Ren ZQ, Yang MR (2023) Analysis of vascular calcification and its influencing factors in elderly diabetic nephropathy patients undergoing maintenance hemodialysis. Geriatr Med Gerontol Health 29(6):1175–1179. 10.3969/j.issn.1008-8296.2023.06.015

[26] Jiang Y (2020) Coronary artery calcification in patients undergoing maintenance hemodialysis: occurrence, progression, and related risk factors. Zhengzhou Univ. 10.27466/d.cnki.gzzdu.2020.003194

[27] Xu J, Shen CJ, Ooi JD et al (2024) Serum sortilin is associated with coronary artery calcification and cardiovascular and cerebrovascular events in maintenance hemodialysis patients. Kidney Dis (Basel, Switzerland) 7(6):503–513 10.1159/000517304 PMC8613630 34901196

[28] Jia FY (2015) Characteristics and determinants of cardiovascular calcification in patients undergoing maintenance hemodialysis. Second Mil Med Univ. 10.7666/d.Y2813079

[29] Xie SY (2022) Analysis of risk factors for coronary artery calcification in patients undergoing maintenance hemodialysis. Southern Med Univ. 10.27003/d.cnki.gojyu.2022.000966

[30] Jiang Y, Wang S, Lai W (2021) Analysis of risk factors for coronary artery calcification in maintenance hemodialysis patients. J Clin Cardiovasc Dis 37(5):428–432. 10.13201/j.issn.1001-1439.2021.05.008

[31] Ran YL, Su JJ, Li XQ et al (2016) Correlation study of serum fibroblast growth factor-23 with coronary artery calcification in patients undergoing maintenance hemodialysis. Southwest Mil Med J 18(4):338–341. 10.3969/j.issn.1672-7193.2016.04.016

[32] Zhou FL, Zhu Z, Lu L (2020) Correlation between coronary artery calcification and serum galectin-3, matrix metalloproteinase-9 levels in patients undergoing maintenance hemodialysis. Guangxi Med J 42(9):1069–1071, 1075. 10.11675/j.issn.0253-4304.2020.09.03

[33] He J, Pan M, Xu M et al (2021) Circulating miRNA-29b and sclerostin levels correlate with coronary artery calcification and cardiovascular events in maintenance hemodialysis patients. Cardiol Res Pract. 10.1155/2021/9208634 34976409 10.1155/2021/9208634 PMC8718313

[34] Shao LN, Li Y, Fan W et al (2021) Analysis of risk factors for coronary artery calcification in diabetic nephropathy patients undergoing maintenance hemodialysis. China Medical Guide 18(20):55–58

[35] Huang H, Zhang AH, Chen J et al (2022) Advances and controversies in vascular calcification research and clinical practice. Acta Physiol Sin 74(6):859–884. 10.13294/j.aps.2022.0102 36594376

[36] Lai AC, Bienstock SW, Sharma R et al (2021) A personalized approach to chronic kidney disease and cardiovascular disease: JACC review topic of the week. J Am Coll Cardiol 77(11):1470–1479. 10.1016/j.jacc.2021.01.028 33736830 10.1016/j.jacc.2021.01.028

[37] Zuo XL, Shang HX (2021) Comparative study of aortic and carotid calcification in patients undergoing hemodialysis and peritoneal dialysis. Chin J Health Care Med Prot 23(2):159–162. 10.3969/j.issn.1674-3245.2021.02.015

[38] Tomikashi K (2022) A comparative study of coronary artery calcification in dialysis patients: diabetic nephropathy group vs. non-diabetic nephropathy group. J Jpn Soc Dial Ther 55(8):485–491. 10.4009/jsdt.55.485

[39] Zhu L, Yang B, Cai M et al (2022) The relationship between post-dialysis volume overload and long-term prognosis in maintenance hemodialysis patients. Chin J Blood Purif 21(3):162–166. 10.3969/j.issn.1671-4091.2022.03.005

[40] Li Q, Li P, Xu Z, Lu Z, Yang C, Ning J (2024) Association of diabetes with cardiovascular calcification and all-cause mortality in end-stage renal disease in the early stages of hemodialysis: a retrospective cohort study. Cardiovasc Diabetol 23(1):259. 10.1186/s12933-024-02318-8 39026232 10.1186/s12933-024-02318-8 PMC11264609

[41] Chen NX et al (2006) High glucose increases the expression of Cbfa1 and BMP-2 and enhances the calcification of vascular smooth muscle cells. Nephrol Dial Transpl 21(12):3435–3442 10.1093/ndt/gfl429 17005530

[42] Maddaloni E, Coraggio L, Amendolara R, Baroni MG, Cavallo MG, Copetti M et al (2023) Association of osteocalcin, osteoprotegerin, and osteopontin with cardiovascular disease and retinopathy in type 2 diabetes. Diabetes Metab Res Rev 39(5):e3632. 10.1002/dmrr.3632 36880127 10.1002/dmrr.3632

[43] Boström KI, Jumabay M, Matveyenko A, Nicholas SB, Yao Y (2011) Activation of vascular bone morphogenetic protein signaling in diabetes mellitus. Circ Res 108(4):446–457. 10.1161/CIRCRESAHA.110.231001 21193740 10.1161/CIRCRESAHA.110.236596 PMC3042480

[44] Lanzer P, Hannan FM, Lanzer JD, Janzen J, Raggi P, Furniss D et al (2021) Medial arterial calcification: JACC state-of-the-art review. J Am Coll Cardiol 78(11):1145–1165. 10.1016/j.jacc.2021.07.027 34503684 10.1016/j.jacc.2021.06.049 PMC8439554

[45] Yan J, Li L, Zhang M, Liu B, Du J, Cui W et al (2018) Circulating bone-specific alkaline phosphatase and abdominal aortic calcification in maintenance hemodialysis patients. Biomark Med 12(11):1231–1239. 10.2217/bmm-2018-0089 30499685 10.2217/bmm-2018-0089

[46] Chronic Kidney Disease Hypertension Management Consensus Expert Group (2023) Chinese expert consensus on the management of hypertension in non-dialysis and dialysis chronic kidney disease patients. Chin J Intern Med 62(7):748–774. 10.3760/cma.j.cn112138-20220909-00670 10.3760/cma.j.cn112138-20220909-00670 37394846

[47] Zhang H, Li G, Yu X et al (2023) Progression of vascular calcification and clinical outcomes in patients receiving maintenance dialysis. JAMA Netw Open 6(5):e2310909. 10.1001/jamanetworkopen.2023.10909 37126347 10.1001/jamanetworkopen.2023.10909 PMC10152309

[48] Xu G, Guo SM, Ge SW (2019) Comprehensive management of serum phosphorus, calcium, and parathyroid hormone in patients with chronic kidney disease. Clin J Nephrol 19(10):715–718. 10.3969/j.issn.1671-2390.2019.10.001

[49] Feng Q, Han XW, Li SX et al (2023) Best evidence summary for the prevention and management of hyperkalemia in maintenance hemodialysis patients. Guangxi Med J 45(23):2893–2900. 10.11675/j.issn.0253-4304.2023.23.16

[50] Jun JE, Lee YB, Lee SE et al (2018) Elevated serum uric acid predicts the development of moderate coronary artery calcification independent of conventional cardiovascular risk factors. Atherosclerosis 272:233–239. 10.1016/j.atherosclerosis.2018.02.014 29482886 10.1016/j.atherosclerosis.2018.02.014

[51] Kang YW, Yang W, Ma SJ et al (2024) Serum levels of sICAM-1, sVCAM-1, and SOD activity in maintenance hemodialysis patients and their relationship with coronary artery calcification. Jilin Univ J Med Ed 000(3):7. 10.13481/j.1671-587X.20240327

[52] Carrillo-López N, Panizo S, Alonso-Montes C et al (2019) High-serum phosphate and parathyroid hormone distinctly regulate bone loss and vascular calcification in experimental chronic kidney disease. Nephrol Dial Transpl. 10.1093/ndt/gfy287 10.1093/ndt/gfy287 30189026

[53] Wu M, Zhang JD, Tang RN et al (2017) Elevated PTH induces endothelial-to- chondrogenic transition in aortic endothelial cells. Am J Physiol Renal Physiol 312(3):F436–F444. 10.1152/ajprenal.00210.2016 27582099 10.1152/ajprenal.00210.2016 PMC5374307

[54] National Clinical Research Center for Kidney Diseases (2019) Summary of the Chinese guidelines for the diagnosis and treatment of chronic kidney disease-mineral and bone disorder. J Nephrol Dial Transpl 28(1):6. 10.3969/j.issn.1006-298X.2019.01.002

[55] Thyroid Surgeon Committee of the Surgeon Branch of the Chinese Medical Doctor Association, Thyroid Disease Committee of the Chinese Research Hospital Association (2021) Chinese expert consensus on surgical clinical practice for secondary hyperparathyroidism in chronic kidney disease (2021 edition). Chin J Pract Surg 41(8):8. 10.19538/j.cjps.issn1005-2208.2021.08.02

[56] Kurozumi A, Nakano K, Yamagata K et al (2019) IL-6 and sIL-6R induce STAT3-dependent differentiation of human VSMCs into osteoblast-like cells through JMJD2B-mediated histone demethylation of RUNX2. Bone. 10.1016/j.bone.2019.04.006 30981888 10.1016/j.bone.2019.04.006

[57] Zhao JL, Zhang T, Min DY, Wu XH, Zhan QN (2020) The relationship between serum sclerostin and coronary artery calcification and cardiovascular events in patients undergoing maintenance hemodialysis. J Nephrol Dial Transpl 29(3):7

[58] Wang Y, Zhang H, Li X et al (2022) Hypertension and coronary artery calcification in dialysis patients: a systematic review and meta-analysis. J Hypertens 40(7):1321–1330. 10.3390/ijms17091481

[59] Harlacher E, Wollenhaupt J, Baaten CC et al (2022) Impact of uremic toxins on endothelial dysfunction in chronic kidney disease: a systematic review. Int J Mol Sci 23(1):531. 10.3390/ijms23010531 35008960 10.3390/ijms23010531 PMC8745705

[60] Li Q, Yao X, Chen J et al (2020) Effects of serum magnesium level on mortality in maintenance hemodialysis patients. Chin J Nephrol 36(11):817–823. 10.3760/cma.j.cn441217-20200105-00144

[61] Jia F (2023) Paying attention to changes in serum magnesium in end-stage renal disease patients undergoing hemodialysis. Chin J Nephrol Dial Transpl 32(3):281–285. 10.3969/j.issn.1006-298X.2023.03.017

[62] Wu H, Liu X (2022) Research progress on the mechanism of magnesium in regulating cardiovascular calcification. Chin J Cardiovasc Med 27(6):513–518. 10.3760/cma.j.cn113805202206/1443918

[63] Zhang J, Xu J, Wang S et al (2014) Effect and mechanism of magnesium ions on phosphorus-induced thoracic aortic calcification in chronic renal failure rats. Chin Gen Pract 17(36):4324–4328. 10.3969/j.issn.1007-9572.2014.36.014

[64] Sakaguchi Y, Fujii N, Shoji T et al (2014) Hypomagnesemia is a significant predictor of cardiovascular and non-cardiovascular mortality in patients undergoing hemodialysis. Kidney Int 85(1):174–181. 10.1038/ki.2013.327 23986148 10.1038/ki.2013.327

[65] Ter BAD, Vervloet MG, De BJHF et al (2020) Magnesium to prevent kidney disease–associated vascular calcification: crystal clear? Nephrol Dial Transpl. 10.1093/ndt/gfaa222 10.1093/ndt/gfaa222 PMC8875474 33374019

[66] Yi Y, Wang Y, Huang W et al (2021) Effects of high-flux hemodialysis on microinflammation, FGF23, and atherosclerosis in elderly maintenance hemodialysis patients. Chin J Gerontol 41(22):5018–5021

[67] Wei X, Lu X, Li X (2024) Analysis of serum PTH and ALP levels in maintenance hemodialysis patients and their association with concurrent cardiovascular events: a clinical study—review of “Diagnosis and Treatment Guidelines for Malignant Hematological Diseases.” Chin J Exp Tradit Med Formulae 30(13):288

[68] Zhao J, Zhang T, Min D, Wu X, Zhan Q (2020) Association of serum sclerostin with coronary artery calcification and cardiovascular events in maintenance hemodialysis patients. Chin J Nephrol Dial Transpl 29(3):208–214. 10.3969/j.issn.1006-298X.2020.03.002

[69] Villa-Bellosta R (2018) Synthesis of extracellular pyrophosphate increases in vascular smooth muscle cells during phosphate-induced calcification. Arterioscler Thromb Vasc Biol 38(9):2137–2147. 10.1161/ATVBAHA.118.311444 30002059 10.1161/ATVBAHA.118.311444

[70] Villa-Bellosta R, Wang X, Millán JL, Dubyak GR, O’Neill WC (2011) Extracellular pyrophosphate metabolism and calcification in vascular smooth muscle. Am J Physiol Heart Circ Physiol 301(1):H61–H68. 10.1152/ajpheart.01020.2010 21490328 10.1152/ajpheart.01020.2010 PMC3129914

