# Supplementary material for: Risk factors for coronary artery calcification in Chinese patients undergoing maintenance hemodialysis: a meta-analysis
Source: Int Urol Nephrol. 2025 May 2;57(10):3307–18. doi: 10.1007/s11255-025-04535-w (PMC12464000; doi:10.1007/s11255-025-04535-w)
Supplement: Supplementary file 1 — Supplementary file1 (DOCX 48 KB) [file 11255_2025_4535_MOESM1_ESM.docx]

**Identification of studies via databases and registers**

**Records identified from*:（n=4963）**

CNKl （n=937)、PubMed(n=1096)、Web of Science(n=552)、Wanfang Data(n=1494)、VIP(n=269)、Ovid(n=195)、Embase(n=66)、CochraneLibrary(n=221)、CMB(sinomed)(n=78)、EBSCO(n=45)

ChiCTR(n=1）, Other gray literature(n=17）

**Identification**

Deletion of duplicates(n=1978)

Read the title and abstract(n =2994 )

Exclude irrelevant literature(n = 2363)

Exclusion of non-human literature(n = 413)

**Screening**

Read the full text(n =218)

**Reports excluded:(n =193 )**

Studies using different outcome indictors (n =129 )

Studies whose full text cannot be acquired (n = 4)

Repeated publications(n =4)

Unsuitable research subjects(n = 50)

Overviews, conferences or abstracts(n =7)

**Included**

**Literature finally included(n =24)**

*Consider, if feasible to do so, reporting the number of records identified from each database or register searched (rather than the total number across all databases/registers).

**If automation tools were used, indicate how many records were excluded by a human and how many were excluded by automation tools.

Source: Page MJ, et al. BMJ 2021;372:n71. doi: 10.1136/bmj.n71.

This work is licensed under CC BY 4.0. To view a copy of this license, visit <https://creativecommons.org/licenses/by/4.0/>
