# Supplementary material for: Risk factors for coronary artery calcification in Chinese patients undergoing maintenance hemodialysis: a meta-analysis
Source: Int Urol Nephrol. 2025 May 2;57(10):3307–18. doi: 10.1007/s11255-025-04535-w (PMC12464000; doi:10.1007/s11255-025-04535-w)
Supplement: Supplementary file 2 — Supplementary file2 (DOCX 34 KB) [file 11255_2025_4535_MOESM2_ESM.docx]

| **Section and Topic** | **Item #** | **Checklist item** | **Location where item is reported** |
| --- | --- | --- | --- |
| **TITLE** | | |  |
| Title | 1 | Identify the report as a systematic review. | Risk Factors Associated with Coronary Artery Calcification in Chinese Patients Undergoing Maintenance Hemodialysis: **A Meta-Analysis**" |
| **ABSTRACT** | | |  |
| Abstract | 2 | See the PRISMA 2020 for Abstracts checklist. | **Abstract**: |
| **INTRODUCTION** | | |  |
| Rationale | 3 | Describe the rationale for the review in the context of existing knowledge. | **Introduction**: |
| Objectives | 4 | Provide an explicit statement of the objective(s) or question(s) the review addresses. | **Abstract (Objective)**: |
| **METHODS** | | |  |
| Eligibility criteria | 5 | Specify the inclusion and exclusion criteria for the review and how studies were grouped for the syntheses. | **Study selection criteria**: |
| Information sources | 6 | Specify all databases, registers, websites, organisations, reference lists and other sources searched or consulted to identify studies. Specify the date when each source was last searched or consulted. | **Literature search strategy**: |
| Search strategy | 7 | Present the full search strategies for all databases, registers and websites, including any filters and limits used. | **2.1 Literature search strategy**: Describes search terms (e.g., "renal dialysis," "coronary artery calcification," "risk factors") and mentions "combination of subject and free-text terms." |
| Selection process | 8 | Specify the methods used to decide whether a study met the inclusion criteria of the review, including how many reviewers screened each record and each report retrieved, whether they worked independently, and if applicable, details of automation tools used in the process. | **2.3 Literature screening and data extraction**: States that two researchers independently screened studies, cross-checked results, and resolved discrepancies with a third researcher. |
| Data collection process | 9 | Specify the methods used to collect data from reports, including how many reviewers collected data from each report, whether they worked independently, any processes for obtaining or confirming data from study investigators, and if applicable, details of automation tools used in the process. | **2.3 Literature screening and data extraction**: Describes data extraction variables (authors, publication year, sample size, etc.) and use of Excel for organization. |
| Data items | 10a | List and define all outcomes for which data were sought. Specify whether all results that were compatible with each outcome domain in each study were sought (e.g. for all measures, time points, analyses), and if not, the methods used to decide which results to collect. | **3.3 Meta-analysis of Factors Influencing Concurrent CAC**: Lists outcomes (e.g., age, diabetes mellitus, biochemical parameters) and specifies inclusion criteria. |
|  | 10b | List and define all other variables for which data were sought (e.g. participant and intervention characteristics, funding sources). Describe any assumptions made about any missing or unclear information. | **Table 1** |
| Study risk of bias assessment | 11 | Specify the methods used to assess risk of bias in the included studies, including details of the tool(s) used, how many reviewers assessed each study and whether they worked independently, and if applicable, details of automation tools used in the process. | **2.4 Evaluation of literature quality**: Describes use of NOS and AHRQ tools, with exclusion of low-quality studies (NOS ≤ 3 or AHRQ < 4). |
| Effect measures | 12 | Specify for each outcome the effect measure(s) (e.g. risk ratio, mean difference) used in the synthesis or presentation of results. | **2.5 Statistical analysis**: States use of OR with 95% CI as effect estimates. |
| Synthesis methods | 13a | Describe the processes used to decide which studies were eligible for each synthesis (e.g. tabulating the study intervention characteristics and comparing against the planned groups for each synthesis (item #5). | **3.3 Meta-analysis of Factors Influencing Concurrent CAC**: Groups studies by sociodemographic, comorbid, and biochemical factors. |
|  | 13b | Describe any methods required to prepare the data for presentation or synthesis, such as handling of missing summary statistics, or data conversions. | **2.5 Statistical analysis**: Mentions sensitivity analyses and subgroup analyses to address heterogeneity. |
|  | 13c | Describe any methods used to tabulate or visually display results of individual studies and syntheses. | **Figures in S1 Appendix** (e.g., S5-S7) and **Table 2** (Heterogeneity Test and Meta-analysis Results). |
|  | 13d | Describe any methods used to synthesize results and provide a rationale for the choice(s). If meta-analysis was performed, describe the model(s), method(s) to identify the presence and extent of statistical heterogeneity, and software package(s) used. | **2.5 Statistical analysis**: Details use of Stata 17.0, fixed/random-effects models, I² statistic, and Q-test for heterogeneity. |
|  | 13e | Describe any methods used to explore possible causes of heterogeneity among study results (e.g. subgroup analysis, meta-regression). | **3.3.2 Subgroup Analyses and Meta-Regression**: Explains subgroup analyses for high-heterogeneity factors and meta-regression for age. |
|  | 13f | Describe any sensitivity analyses conducted to assess robustness of the synthesized results. | **3.3.4 Sensitivity analysis**: Details "one-by-one exclusion" method and comparison of fixed/random-effects models. |
| Reporting bias assessment | 14 | Describe any methods used to assess risk of bias due to missing results in a synthesis (arising from reporting biases). | **3.3.4 Publication bias**: Uses funnel plots, Begg’s test, and Egger’s test to evaluate bias. |
| Certainty assessment | 15 | Describe any methods used to assess certainty (or confidence) in the body of evidence for an outcome. | **3.1 Literature search and screening**: References **Figure 1** (flow chart) showing screening steps from 4,981 records to 24 included studies. |
| **RESULTS** | | |  |
| Study selection | 16a | Describe the results of the search and selection process, from the number of records identified in the search to the number of studies included in the review, ideally using a flow diagram. | **Table 1**: Lists all included studies with details (author, year, sample size, influencing factors, quality scores). |
|  | 16b | Cite studies that might appear to meet the inclusion criteria, but which were excluded, and explain why they were excluded. | **Table 1** (NOS/AHRQ column): Reports quality scores (e.g., NOS 7-9 for cohort studies). |
| Study characteristics | 17 | Cite each included study and present its characteristics. | **Table 2** and **S1 Appendix S5-S7**: Provide ORs, 95% CIs, and heterogeneity metrics for each factor. |
| Risk of bias in studies | 18 | Present assessments of risk of bias for each included study. | **3.2 Characteristics and quality assessment**: Summarizes study types, sample sizes, and quality scores. |
| Results of individual studies | 19 | For all outcomes, present, for each study: (a) summary statistics for each group (where appropriate) and (b) an effect estimate and its precision (e.g. confidence/credible interval), ideally using structured tables or plots. | **3.3.1 Meta-analysis results**: Reports pooled ORs and 95% CIs for significant factors (e.g., diabetes: OR=2.32). |
| Results of syntheses | 20a | For each synthesis, briefly summarise the characteristics and risk of bias among contributing studies. | **3.3.2 Subgroup Analyses and Meta-Regression**: Discusses sources of heterogeneity (e.g., study design differences). |
|  | 20b | Present results of all statistical syntheses conducted. If meta-analysis was done, present for each the summary estimate and its precision (e.g. confidence/credible interval) and measures of statistical heterogeneity. If comparing groups, describe the direction of the effect. | **3.3.4 Sensitivity analysis**: Describes stability of results after excluding specific studies. |
|  | 20c | Present results of all investigations of possible causes of heterogeneity among study results. | **3.3.4 Publication bias**: Reports Egger’s test results (e.g., publication bias for age, dialysis duration). |
|  | 20d | Present results of all sensitivity analyses conducted to assess the robustness of the synthesized results. | **4 Discussion**: Compares findings with prior studies (e.g., Hui et al.[35]) and discusses mechanisms (e.g., age-related vascular changes). |
| Reporting biases | 21 | Present assessments of risk of bias due to missing results (arising from reporting biases) for each synthesis assessed. | **4 Discussion (Limitations)**: Mentions heterogeneity in existing studies and potential publication bias. |
| Certainty of evidence | 22 | Present assessments of certainty (or confidence) in the body of evidence for each outcome assessed. | **4 Discussion**: Notes limitations in study designs (e.g., variability in sample sizes) and methodological heterogeneity. |
| **DISCUSSION** | | |  |
| Discussion | 23a | Provide a general interpretation of the results in the context of other evidence. | **1.1 Registration**: States PROSPERO registration number (CRD42024606240). |
|  | 23b | Discuss any limitations of the evidence included in the review. | **3.3 Meta-analysis of Factors Influencing Concurrent CAC**: Groups studies by sociodemographic, comorbid, and biochemical factors. |
|  | 23c | Discuss any limitations of the review processes used. | **2.5 Statistical analysis**: Mentions sensitivity analyses and subgroup analyses to address heterogeneity. |
|  | 23d | Discuss implications of the results for practice, policy, and future research. | **Figures in S1 Appendix** (e.g., S5-S7) and **Table 2** (Heterogeneity Test and Meta-analysis Results). |
| **OTHER INFORMATION** | | |  |
| Registration and protocol | 24a | Provide registration information for the review, including register name and registration number, or state that the review was not registered. | **3.3.2 Subgroup Analyses and Meta-Regression**: Explains subgroup analyses for high-heterogeneity factors and meta-regression for age. |
|  | 24b | Indicate where the review protocol can be accessed, or state that a protocol was not prepared. | **3.3.4 Sensitivity analysis**: Details "one-by-one exclusion" method and comparison of fixed/random-effects models. |
|  | 24c | Describe and explain any amendments to information provided at registration or in the protocol. | **3.3.4 Publication bias**: Uses funnel plots, Begg’s test, and Egger’s test to evaluate bias. |
| Support | 25 | Describe sources of financial or non-financial support for the review, and the role of the funders or sponsors in the review. | **3.1 Literature search and screening**: References **Figure 1** (flow chart) showing screening steps from 4,981 records to 24 included studies. |
| Competing interests | 26 | Declare any competing interests of review authors. | **2.2 Study selection criteria**: Lists exclusion criteria (e.g., low-quality studies, non-Chinese literature). Specific excluded studies are not listed but implied by the screening process. |
| Availability of data, code and other materials | 27 | Report which of the following are publicly available and where they can be found: template data collection forms; data extracted from included studies; data used for all analyses; analytic code; any other materials used in the review. | **Table 1**: Lists all included studies with details (author, year, sample size, influencing factors, quality scores). |

*From:*  Page MJ, McKenzie JE, Bossuyt PM, Boutron I, Hoffmann TC, Mulrow CD, et al. The PRISMA 2020 statement: an updated guideline for reporting systematic reviews. BMJ 2021;372:n71. doi: 10.1136/bmj.n71
