# Supplementary material for: Risk factors for coronary artery calcification in Chinese patients undergoing maintenance hemodialysis: a meta-analysis
Source: Int Urol Nephrol. 2025 May 2;57(10):3307–18. doi: 10.1007/s11255-025-04535-w (PMC12464000; doi:10.1007/s11255-025-04535-w)
Supplement: Supplementary file 3 — Supplementary file3 (DOCX 671 KB) [file 11255_2025_4535_MOESM3_ESM.docx]

# **S1 Appendix**

**S1 Table: Search strategy：**

| **PubMed (Initial search August 2024, Updated October 2024)** |
| --- |
| (((Renal Dialysis[Mesh]) OR (((((((hemodialysis[Title/Abstract])) OR (extracorporeal dialysis[Title/Abstract])) OR (renal dialysis[Title/Abstract])) OR (blood dialysis[Title/Abstract])) OR (maintenance hemodialysis[Title/Abstract])) OR (MHD[Title/Abstract]))) AND ((((((Vascular Calcification[Title/Abstract]) OR (vascular calcinoses[Title/Abstract])) OR (coronary calcification[Title/Abstract])) OR (calcification of blood vessels[Title/Abstract])) OR (coronary arterycalcification[Title/Abstract])) OR (Vascular Calcification[Mesh]))) AND ((Risk Factors[Mesh]) OR ((((((factor[Title/Abstract]) OR (influence factors*[Title/Abstract])) OR (associated factors[Title/Abstract])) OR (relevant factors*[Title/Abstract])) OR (risk factors[Title/Abstract])) OR (affecting factor[Title/Abstract])) OR (dangerous factor[Title/Abstract])) OR (reason[Title/Abstract]))) |
| **Web of Science Core Collection (Initial search August 2024, Updated October 2024)** |
| TS=(hemodialysis OR extracorporeal dialysis OR renal dialysis OR blood dialysis OR maintenance hemodialysis OR MHD ) AND TS=(Vascular Calcification OR vascular calcinosis OR coronary calcification OR calcification of blood vessels OR coronary artery calcification) AND TS=(factor OR influence factors OR associated factors OR relevant factors OR risk factors OR affecting factor OR dangerous factor OR reason) AND Address=(China OR Taiwan OR Hong Kong OR Macao OR Chinese OR province OR city) |
| **Chinese Biomedical Literature Database (Initial search August 2024, Updated October 2024)** |
| ( "透析"[常用字段:智能] OR "尿毒症"[常用字段:智能] OR "终末期肾病"[常用字段:智能] OR "肾衰竭"[常用字段:智能] OR "血液透析"[常用字段:智能] OR "维持性血液透析"[常用字段:智能] OR "体外透析 OR血液净化"[常用字段:智能]) AND( "冠脉钙化"[常用字段:智能] OR "血管钙化"[常用字段:智能] OR "冠状动脉钙化"[常用字段:智能]) AND( "影响因素"[常用字段:智能] OR "相关因素"[常用字段:智能] OR "危险因素"[常用字段:智能] OR "预测因素"[常用字段:智能] OR "病因"[常用字段:智能] OR "有关因素"[常用字段:智能]) AND( "中国"[常用字段:智能] OR "台湾"[常用字段:智能] OR "香港"[常用字段:智能] OR "澳门"[常用字段:智能] OR "省"[常用字段:智能] OR "市"[常用字段:智能] OR "地区"[常用字段:智能]) |
| **EBSCO(Initial search August 2024, Updated October 2024)** |
| SU=( hemodialysis or haemodialysis or dialysis or extracorporeal dialysis or renal dialysis or blood dialysis or maintenance hemodialysis or MHD ) AND SU=( vascular calcification or vascular calcinoses or coronary calcification or calcification of blood vessels or coronary arterycalcification ) AND SU=( influence factors or associated factors or relevant factors or risk factors or affecting factor or dangerous factor or reason or contributing factors or predisposing factors ) AND AU=( China OR Taiwan OR Hong Kong OR Macao OR Chinese OR province OR prefecture-level city ) |
| **The Cochrane Library (Initial search August 2024, Updated October 2024)** |
| (hemodialysis):ti,ab,kw OR (haemodialysis):ti,ab,kw OR (dialysis):ti,ab,kw OR (extracorporeal dialysis):ti,ab,kw OR (renal dialysis):ti,ab,kw OR (blood dialysis):ti,ab,kw OR (maintenance hemodialysis):ti,ab,kw OR (MHD):ti,ab,kw AND (risk factors):ti,ab,kw OR (influence factors):ti,ab,kw OR (associated factors):ti,ab,kw OR (relevant factors):ti,ab,kw OR (affecting factor):ti,ab,kw OR (dangerous facto):ti,ab,kw OR (relevant factors):ti,ab,kw OR (dangerous factor):ti,ab,kw OR (contributing factors):ti,ab,kw OR (predisposing factors):ti,ab,kw AND (Vascular Calcification):ti,ab,kw OR (vascular calcinoses):ti,ab,kw OR (coronary calcification):ti,ab,kw OR (calcification of blood vessels):ti,ab,kw OR (coronary arterycalcification):ti,ab,kw |
| **Embase (Initial search August 2024, Updated October 2024)** |
| SU ( hemodialysis or haemodialysis or dialysis or extracorporeal dialysis or renal dialysis or blood dialysis or maintenance hemodialysis or MHD ) AND SU ( vascular calcification or vascular calcinoses or coronary calcification or calcification of blood vessels or coronary arterycalcification ) AND SU ( risk factors or contributing factors or predisposing factors or predictor or cause or vulnerability factors or factor or reason ) AND AU ( China OR Taiwan OR Hong Kong OR Macao OR Chinese OR province OR prefecture-level city ) |
| **Ovid(Initial search August 2024, Updated October 2024)** |
| Subject HeadingWord=(vascular calcification or vascular calcinoses or coronary calcification or calcification of blood vessels or coronary arterycalcification) and Subject HeadingWord=(risk factors or contributing factors or predisposing factors or predictor or cause or vulnerability factors or prevalenceinfluence factors or associated factors or relevant factors or risk factors or affecting factor or dangerous factor or reason or contributing factors or predisposing factors) and Subject HeadingWord=(MHD or Renal Dialysis or Dialysis, Renal or Renal dialysis or Hemodialysis or hemodialysis or Dialysis, Extracorporeal or dialysis, Extracorporeal or Extracorporeal dialysis or Extracorporeal Dialysis or peritoneal dialysis or Dialysis or blood purification). |
| **China National Knowledge Infrastructure (Initial search August 2024, Updated October 2024)** |
| SU%=（动脉钙化+血管钙化+冠状动脉钙化） AND SU%=（透析+血液透析+体外透析+肾透析+血透+维持性血液透析） AND SU%=（影响因素+相关因素+危险因素+风险因素+相关） |
| **Wanfang database (Initial search August 2024, Updated October 2024)** |
| 主题:(动脉钙化 or 血管钙化 or 冠状动脉钙化）AND 主题:(血液透析 or 体外透析 or 肾透析 or 血透 or 维持性血液透析) AND 主题:(影响因素 or 相关因素 or 危险因素 or 风险因素 or 相关 or 剖面分析) |
| **Vip citation database (Initial search August 2024, Updated October 2024)** |
| M=（动脉钙化 OR 血管钙化 OR 冠状动脉钙化） AND M=（透析 OR 血液透析 OR 体外透析 OR 肾透析 OR 血透 OR 维持性血液透析） AND M=（影响因素 OR 相关因素 OR 危险因素 OR 风险因素 OR 相关） |

# **S2 Table: Results of quality assessment of cross-sectional studies on the criteria of AHRQ**

| Study | 1 | 2 | 3 | 4 | 5 | 6 | 7 | 8 | 9 | 10 | 11 | score |
| --- | --- | --- | --- | --- | --- | --- | --- | --- | --- | --- | --- | --- |
| Cheng 2019 | 1 | 1 | 1 | 1 | 0 | 1 | 0 | 1 | 0 | 1 | 0 | 7 |
| Ran 2016 | 1 | 1 | 1 | 1 | 0 | 1 | 0 | 1 | 0 | 1 | 0 | 8 |
| Zhou2020 | 1 | 1 | 1 | 1 | 0 | 1 | 1 | 1 | 0 | 1 | 0 | 9 |
| Cui 2015 | 1 | 1 | 1 | 1 | 1 | 1 | 0 | 0 | 1 | 1 | 0 | 8 |
| Dong 2024 | 1 | 1 | 1 | 1 | 1 | 1 | 0 | 1 | 1 | 1 | 0 | 9 |
| Chen 2020 | 1 | 1 | 1 | 1 | 0 | 1 | 1 | 0 | 1 | 1 | 0 | 8 |
| Yang 2017 | 1 | 1 | 1 | 1 | 1 | 1 | 0 | 1 | 1 | 1 | 0 | 9 |
| Cui2024 | 1 | 1 | 1 | 1 | 1 | 1 | 1 | 0 | 0 | 1 | 0 | 8 |
| Chen 2017 | 1 | 1 | 1 | 1 | 1 | 1 | 0 | 0 | 1 | 1 | 0 | 8 |
| Zhou 2024 | 1 | 1 | 1 | 1 | 1 | 1 | 0 | 0 | 0 | 1 | 0 | 7 |
| Xiong 2024 | 1 | 1 | 1 | 1 | 1 | 1 | 0 | 0 | 1 | 1 | 0 | 8 |
| Hu 2022 | 1 | 1 | 1 | 1 | 1 | 1 | 1 | 1 | 0 | 1 | 0 | 9 |
| Guo 2023 | 1 | 1 | 1 | 1 | 1 | 1 | 0 | 1 | 1 | 1 | 0 | 9 |

**S3 Table: Results of quality assessment of cohort studies on NOS**

| Study | Selection | | | | Comparability | | Exposure/Outcome | | score | |  |
| --- | --- | --- | --- | --- | --- | --- | --- | --- | --- | --- | --- |
|  | 1 | 2 | 3 | 4 | 5A | 5B | 6 | 7 | 8 |  | |
| Kang 2023 | 1 | 1 | 1 | 1 | 0 | 1 | 0 | 1 | 0 | 6 | |
| Lai 2022 | 1 | 1 | 1 | 1 | 1 | 1 | 0 | 1 | 0 | 7 | |
| Shao 2021 | 1 | 1 | 1 | 1 | 1 | 1 | 1 | 1 | 0 | 8 | |
| Fan 2023 | 1 | 1 | 1 | 1 | 1 | 1 | 1 | 1 | 0 | 8 | |

**S4 Table: Results of quality assessment of case-control studies on NOS**

| **Study** | **Selection** | | | | **Comparability** | | **Exposure/Outcome** | | | **score** |
| --- | --- | --- | --- | --- | --- | --- | --- | --- | --- | --- |
|  | **1** | **2** | **3** | **4** | **5A** | **5B** | **6** | **7** | **8** |  |
| Lei2023 | 1 | 1 | 1 | 1 | 1 | 0 | 1 | 1 | 0 | 7 |
| He 2023 | 1 | 1 | 1 | 1 | 1 | 1 | 1 | 1 | 0 | 8 |
| Jiang 2020 | 1 | 1 | 1 | 1 | 1 | 0 | 1 | 1 | 0 | 7 |
| Xu 2021 | 1 | 1 | 1 | 1 | 1 | 1 | 1 | 1 | 0 | 8 |
| Jia2015 | 1 | 1 | 1 | 1 | 1 | 1 | 1 | 1 | 0 | 8 |
| Xie 2022 | 1 | 1 | 1 | 1 | 1 | 1 | 1 | 1 | 0 | 8 |
| Jiang 2021 | 1 | 1 | 1 | 1 | 1 | 0 | 1 | 0 | 0 | 6 |

# **S5 Fig: The forest plot of sociodemographic factors**

|  |  |
| --- | --- |
| **age（P＜0.001）** | **age≥60（P＜0.001）** |
|  |  |
| **Duration of dialysis treatment（P＜0.001）** |  |

**S6 Fig: The forest plot of complications**

|  |  |
| --- | --- |
| **hypertension（P＜0.001）** | **diabetes mellitus（P＜0.001）** |

**[S7 Fig: The forest plot of biochemical factors](#_Toc134190903)**

|  |  |
| --- | --- |
| **phosphorus（P＜0.001）** | **calcium（P＜0.001）** |
|  |  |
| **hs-CRP（P＜0.001）** | **FGF-23（P＜0.001）** |
|  |  |
| **iPTH（P＜0.001）** | **SOST（P＜0.001）** |
|  |  |
| **magnesium（P=0.324）** | **ALP（P＜0.001）** |

**S8 Fig:Subgroup analysis**

|  |  |  |
| --- | --- | --- |
| **Age（Type）** | **Age（Area）** | **Age（Year）** |
|  |  |  |
| **Length of dialysis treatment（Type）** | **Length of dialysis treatment（Area）** | **Length of dialysis treatment（Year）** |
|  |  |  |
| **diabetes mellitus（Type）** | **diabetes mellitus（Area）** | **diabetes mellitus（Year）** |
|  |  |  |
| **SOST（Type）** | **SOST（Area）** | **SOST（Year）** |
|  |  |  |
| **FGF-23（Type）** | **FGF-23（Area）** | **FGF-23（Year）** |
|  |  |  |
| **ALP（Type）** | **ALP（Area）** | **ALP（Year）** |
|  |  |  |
| **hypertension（Type）** | **hypertension（Area）** | **hypertension（Year）** |

# **S9 Fig: The sensitivity analysis plots with meta-analysis**

|  |  |
| --- | --- |
| **Age** | **age≥60** |
|  |  |
| **Duration of dialysis treatment** | **diabetes mellitus** |
|  |  |
| **iPTH** | **SOST** |
|  |  |
| **FGF-23** | **ALP** |

**S10 Fig:publication bias**

| (egger) | (begg) |
| --- | --- |
|  |  |
| **Age** | |
|  |  |
| **IPTH** | |
|  |  |
| **Length of dialysis treatment** | |
|  |  |
| **blood phosphorus** | |

**S11 Fig:funnel plot**

|  |  |
| --- | --- |
| **Age** | **IPTH** |
|  |  |
| **Length of dialysis treatment** | **blood phosphorus** |
